# Texture analysis improves lung-tissue segmentation on high-resolution computed tomography in COVID-19

**DOI:** 10.3389/fradi.2025.1694478

**Published:** 2025-12-05

**Authors:** Mazin Abdalla Hassib, Mohamed E. M. Garelnabi, Qurashi Mohamed Ali, Amjad Rashed Alyahyawi, Mamdouh Saud Al-enezi, Mohammed Salih, Ahmed Babikir Abdalla Hasieb

**Affiliations:** 1 Collage of Applied Medical Sciences - Diagnostic Radiology Department, University of Hail, Ha'il, Saudi Arabia; 2 Faculty of Medicine and Surgery, National University - Sudan, Khartoum, Sudan; 3 WeCare Hospital Dammam, Dammam, Saudi Arabia

**Keywords:** high-resolution computed tomography, COVID-19, texture analysis, lung tissue segmentation, ground-glass opacity, quantitative CT

## Abstract

**Background:**

The accurate separation of lung parenchyma, ground-glass opacity (GGO), and intrapulmonary vessels on high-resolution computed tomography (HRCT) in coronavirus disease 2019 (COVID-19) is challenging.

**Methods:**

We conducted a cross-sectional study that analyzed 530 adults (20–40 years) with RT-PCR-confirmed COVID-19. For texture modeling, we sampled 597 regions of interest (ROIs) representing parenchyma, GGO, and intrapulmonary vessels. Region-of-interest-labeled HRCT patches representing parenchyma, GGO, and vessels were analyzed using first- and second-order texture features that were computed across different square window sizes (5 × 5–20 × 20 pixels). Feature selection with stepwise linear discriminant analysis yielded a three-class classifier. The primary endpoint was overall classification accuracy, with the secondary endpoints including the effect of window size and identification of the most informative features.

**Results:**

The 20 × 20-pixel window produced the highest performance, with an overall accuracy of 88.6%. Five co-occurrence-based features (average difference, inverse difference moment, co-occurrence matrix standard deviation, sum entropy, and information correlation measure 1) were the most discriminative; the majority of the errors occurred at tissue boundaries where patches spanned mixed voxels.

**Conclusion:**

Texture-based feature extraction achieved 88.6% ROI-level accuracy and can serve as a supplementary tool during radiological interpretation of chest CT.

## 1 Introduction

High-resolution computed tomography (HRCT) is essential for diagnosing coronavirus disease 2019 (COVID-19) pneumonia, where ground-glass opacity (GGO) and related patterns predominate; however, differentiating subtle GGO from normal aerated parenchyma and intrapulmonary vessels remains challenging at the voxel level, especially at the lesion margins. The accurate separation of these tissue types can support reporting consistency and quantitative burden estimates (1).

Texture analysis, a component of radiomics, quantifies spatial intensity variation using first- and second-order statistics [e.g., grey-level co-occurrence matrix (GLCM)] and provides interpretable descriptors that complement visual reading. Prior reviews have summarized its clinical applications and common pitfalls, emphasizing acquisition/reconstruction dependence and the need for transparent, reproducible pipelines (2, 3). Methodological standards such as the Image Biomarker Standardization Initiative (IBSI) further promote comparability across software and studies (4).

In chest CT for COVID-19 specifically, texture-based approaches have shown utility for differentiation and risk stratification, for example, distinguishing COVID-19 interstitial pneumonia from other interstitial pneumonias and identifying patients at risk of subsequent fibrotic change (5, 6).

Recent years have seen the application of AI-based segmentation models to COVID-19 HRCT, including architectures such as U-Net, nnU-Net, and Transformer-based designs such as TransUNet and Swin-Unet. These models have demonstrated notable improvements in automated lesion detection and boundary delineation (7–10).

Despite this progress, relatively few studies have directly examined tissue-class separation, i.e., parenchyma vs. GGO vs. intrapulmonary vessels, in HRCT using purely statistical (non-AI) texture features, while also probing how analysis window size influences discrimination and boundary-related error. Addressing this gap could yield practical, interpretable cues that will supplement radiologists’ assessments in everyday cardiothoracic imaging.

In this study, we evaluated whether first- and second-order texture features (with emphasis on GLCM statistics), combined with stepwise linear discriminant analysis, can separate normal parenchyma, GGO, and intrapulmonary vessels on HRCT in adults with COVID-19. We compare their performance across different window sizes (5 × 5–20 × 20 pixels), report the overall and class-wise accuracy, and characterize boundary-related errors, aiming for an applied, reproducible quantitative-CT workflow that augments clinical interpretation rather than developing a standalone artificial intelligence (AI) system.

## 2 Materials and methods

### 2.1 Study design and ethics

We conducted a cross-sectional analytic study following approval from the National University Research Ethical Committee (NU-REC; Approval No. NU-REC/01-04-02). All procedures adhered to the relevant guidelines and regulations. Written informed consent for participation and publication was obtained from all participants using a study-specific consent form. To protect confidentiality, all direct identifiers were removed prior to the analysis. The Sex and Gender Equity in Research (SAGER) guidelines were followed; sex was recorded according to the participants’ clinical records.

### 2.2 Setting and participants

HRCT examinations were retrieved from the radiology picture archiving and communication system (PACS) of Al-Raqi University Hospital. Eligible cases were adults aged 20–40 years with RT-PCR-confirmed COVID-19 and radiological reports consistent with COVID-19 pneumonia on HRCT. Patients with normal lungs or diffuse interstitial lung disease (DILD) were excluded. The final cohort comprised 530 male and female participants.

### 2.3 CT acquisition

HRCT series were acquired according to departmental protocols. For reproducibility, we report the following parameters: scanner: SOMATOM go.Now® (Siemens Healthineers, Germany); without IV contrast; end-inspiration breath-hold; kV: 120; rotation time: 0.5 s; pitch: 1.0; matrix: 512 × 512; slice thickness: 1.0–1.5 mm; and iterative reconstruction. The images were exported in the Digital Imaging and Communications in Medicine (DICOM) format. Windowing for region of interest (ROI) placement used the standard lung settings.

### 2.4 ROI annotation

ROI annotation was performed by the lead author and a trained research team after a structured training and calibration program. ROIs were placed exclusively on axial HRCT lung-window images using the standard lung display, in line with recommended chest CT practice (11). The operational definitions followed the Fleischner Society’s Glossary, with GGO defined as hazy increased attenuation with preservation of underlying bronchial and vascular margins (12). Normal lung parenchyma was defined as aerated lung without visible vessels/bronchi, fissures, and artifacts, and minimal partial-volume effects and structural contamination (11). Intrapulmonary vessels were identified as tubular, branching high-attenuation structures in continuity with the central pulmonary vasculature (12). The ROIs were finalized by consensus after a structured training and calibration process; interreader agreement was not measured.

### 2.5 Bias minimization

To limit analysis bias, all the DICOM data were anonymized; the annotators were blinded to computed features and all model outputs; and ROI selection was completed before any modeling to avoid data leakage (“peeking”), consistent with current guidance on standardization in radiomics (4).

### 2.6 Texture feature extraction

Texture features were computed from each labeled ROI using Interactive Data Language software (IDL; version 2.1, NV5 Geospatial, FL, USA). Square analysis windows 5 × 5, 10 × 10, 15 × 15, and 20 × 20 pixels in size were used. Second-order statistics were derived from the GLCM (also termed spatial grey-level dependence, SGLD) computed with an interpixel distance of 1 and orientation of 0°. The feature set included standard GLCM measures (e.g., average difference, inverse difference moment, co-occurrence-matrix standard deviation, sum entropy, and information correlation measure 1). First-order (histogram-based) descriptors were also available for the analysis.

### 2.7 Feature selection and statistical classification

**Figure 1:**
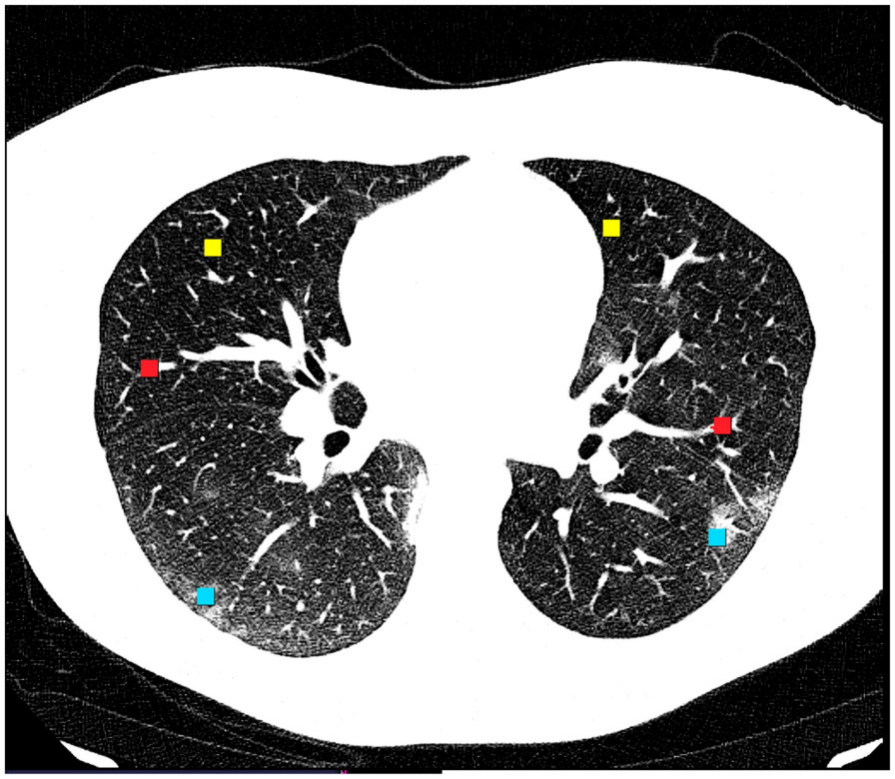
Examples of the regions of interest on an axial HRCT lung-window image illustrating the three tissue classes. Blue squares denote ground-glass opacity (GGO), red squares denote intrapulmonary vessels, and yellow squares denote normal lung parenchyma.

Feature selection employed stepwise linear discriminant analysis (LDA) to identify a compact subset of texture features with high class separability. The selected features trained a three-class classifier to distinguish between normal lung parenchyma, GGO, and intrapulmonary vessels (Figure 1). Each feature vector inherited the class label of its source ROI. Window-size effects (5 × 5 to 20 × 20 pixels) were compared descriptively.

The dataset comprised 530 patients and 597 labeled ROIs (parenchyma = 198; GGO = 201; intrapulmonary vessels = 198), providing roughly balanced class sizes. The stepwise LDA retained a small, interpretable feature set (e.g., average difference, inverse difference moment, GLCM standard deviation, sum entropy, and information correlation measure 1), limiting dimensionality relative to observations.

To mitigate overfitting and prevent patient-level leakage, all the modeling steps, feature selection, and classifier fitting were embedded within patient-level, stratified k-fold cross-validation, and out-of-fold predictions were aggregated to estimate the performance. This yielded metrics based only on the data that were not used for fitting in each fold. Given that there were ∼200 ROIs per class and a small final feature set, the observations-to-parameters ratio comfortably exceeded common heuristics for linear classifiers.

#### 2.7.1 Validation strategy

Performance was estimated using patient-level, stratified k-fold cross-validation with out-of-fold aggregation. The per-class sensitivity, specificity, and macroaveraged F1 values are reported in Supplementary Table S1.

## 3 Result

**Figure 2:**
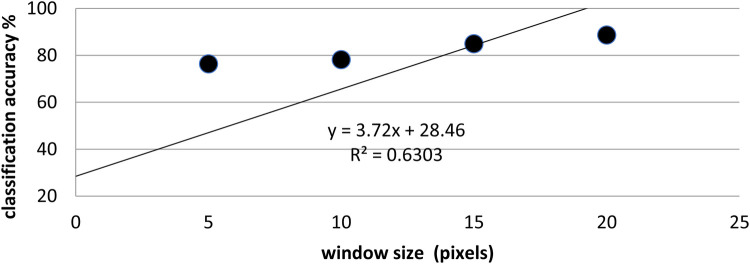
Classification accuracy across analysis-window sizes (5 × 5, 10 × 10, 15 × 15, and 20 × 20 pixels). Accuracy increases monotonically with window size and peaks at 20 × 20 pixels.

In total, 597 labeled ROIs were included in the analysis. The textural features extracted using a 20 × 20-pixel window demonstrated the highest classification accuracy (Figure 2), due to the window size being sufficiently large to capture and quantify the textural features within the defined area. This larger window size enabled a more comprehensive analysis of the global texture characteristics as opposed to a confined or restricted area, contributing to improved classification accuracy.

Classification accuracy across the analysis window sizes (5 × 5, 10 × 10, 15 × 15, and 20 × 20 pixels) is shown in Figure 2. Accuracy increased consistently with window size, peaking at 20 × 20 pixels. The stepwise LDA identified five of 15 texture features as the most discriminatory for the average difference, inverse difference moment, GLCM standard deviation, sum entropy, and information correlation measure 1. Each showed a strong correlation with the class labels and no collinearity.

**Figure 3:**
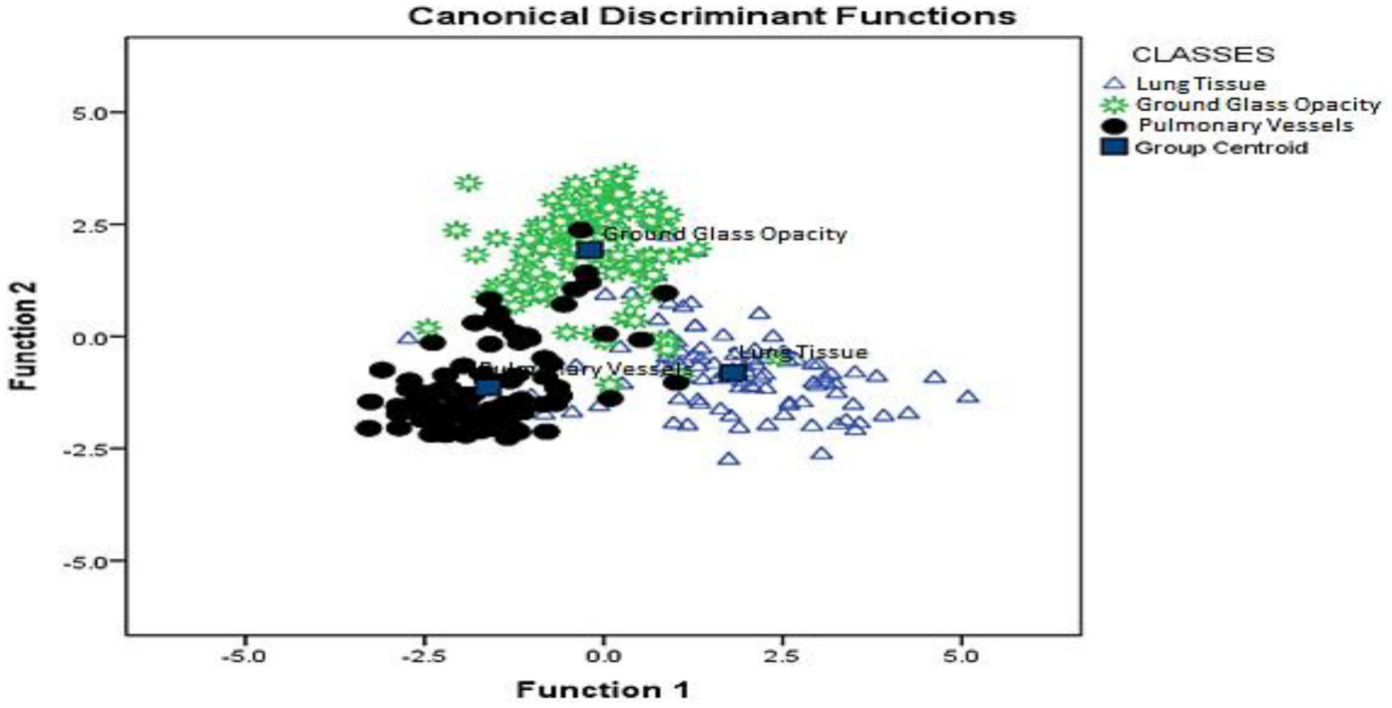
Canonical discriminant functions (linear discriminant analysis) showing ROI distributions by class, i.e., parenchyma, ground-glass opacity (GGO), and intrapulmonary vessels, with class centroids. The plot corresponds to the 20 × 20-pixel window; the cluster overlap qualitatively mirrors the misclassification patterns seen in the ROI-level confusion matrix (Table 1).

These features enabled clear separation among the three classes (parenchyma, GGO, and intrapulmonary vessels), as illustrated in Figure 3, achieving an overall accuracy of 88.6% (529/597) with a binomial 95% CI of 85.8%–90.9%. This was derived from the aggregated out-of-fold predictions. Table 1 summarizes the corresponding confusion matrix.

**Table 1:** Confusion matrix for the three-class ROI-level classification (stepwise LDA; 20 × 20-pixel window).

Class		Predicted group membership			Total
		Lung parenchyma	GGO	Intrapulmonary vessels	
Count	Lung parenchyma	170		1,414	14
	GGO	10	181	10	201
	Intrapulmonary vessels	4	16	178	198
%	Lung parenchyma	85.9	7.1	7.1	100.0
	GGO	5.0	90.0	5.0	100.0
	Intrapulmonary vessels	2.0	8.1	89.9	100.0

Row percentages shown. Overall accuracy: 88.6% (529/597).

GGO demonstrated the highest class-wise accuracy, reflecting its distinct hyperdense textural characteristics compared with the iso-to-hypodense profiles of the lung parenchyma and intrapulmonary vessels. The per-class sensitivity, specificity, and macro-F1 values are provided in Supplementary Table S1.

**Figure 4:**
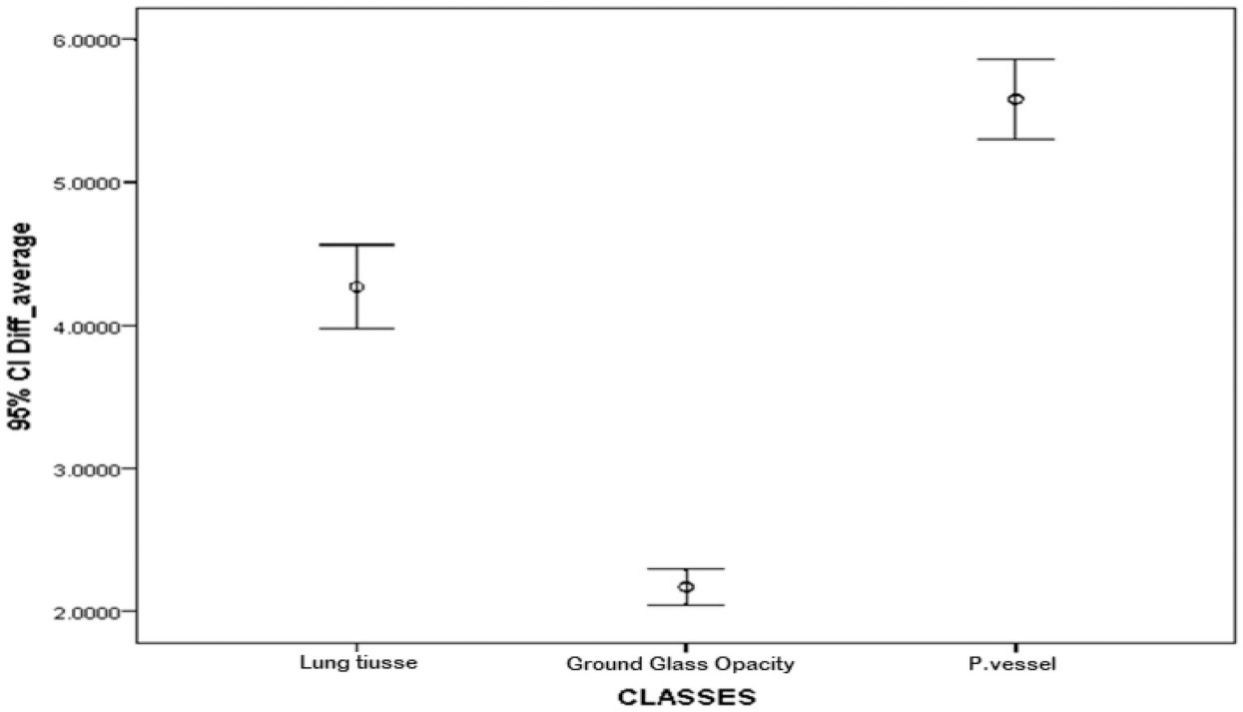
Error-bar plot (mean ± 95% CI) for the average difference texture feature across classes, i.e., parenchyma, ground-glass opacity (GGO), and intrapulmonary vessels, computed at the ROI level (20 × 20-pixel window). The values are the lowest for GGO, intermediate for parenchyma, and highest for intrapulmonary vessels, indicating between-class separation.

**Figure 5:**
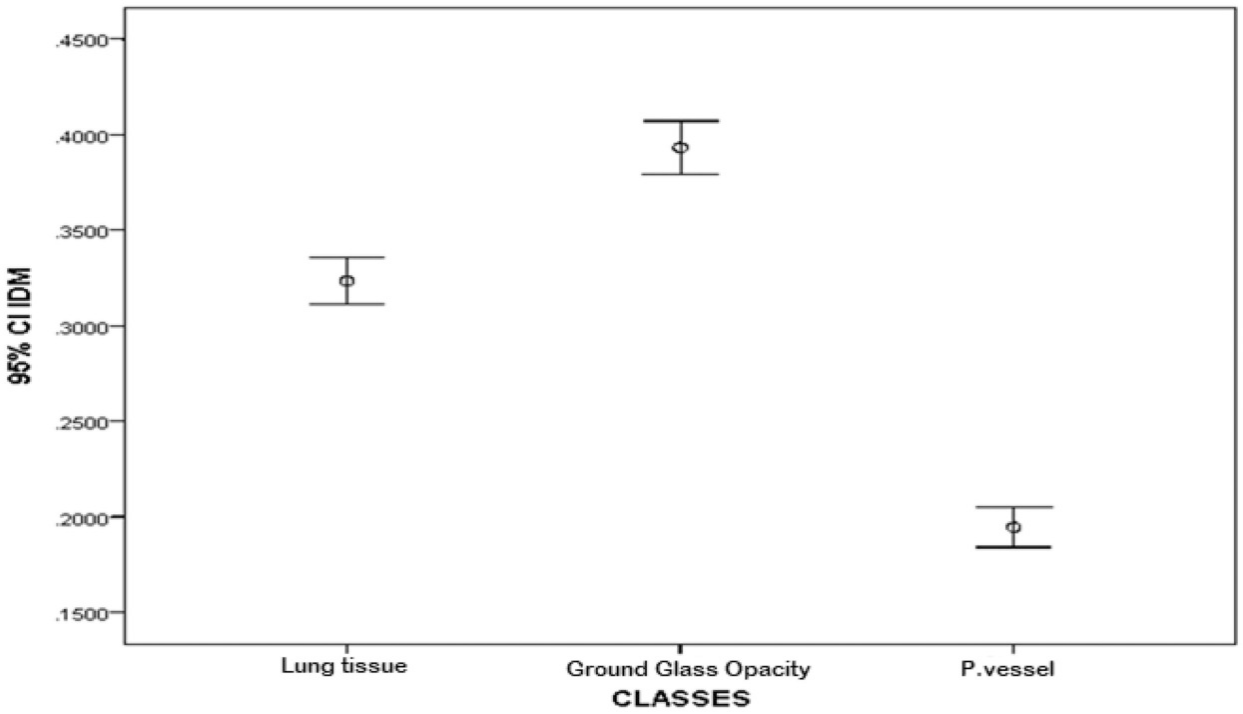
Error-bar plot (mean ± 95% CI) for the inverse difference moment (IDM) texture feature by class, i.e., parenchyma, ground-glass opacity (GGO), and intrapulmonary vessels, computed at the ROI level (20 × 20-pixel window). The IDM is the highest for GGO, intermediate for parenchyma, and lowest for intrapulmonary vessels, indicating complementary separation to Figure 4.

**Figure 6:**
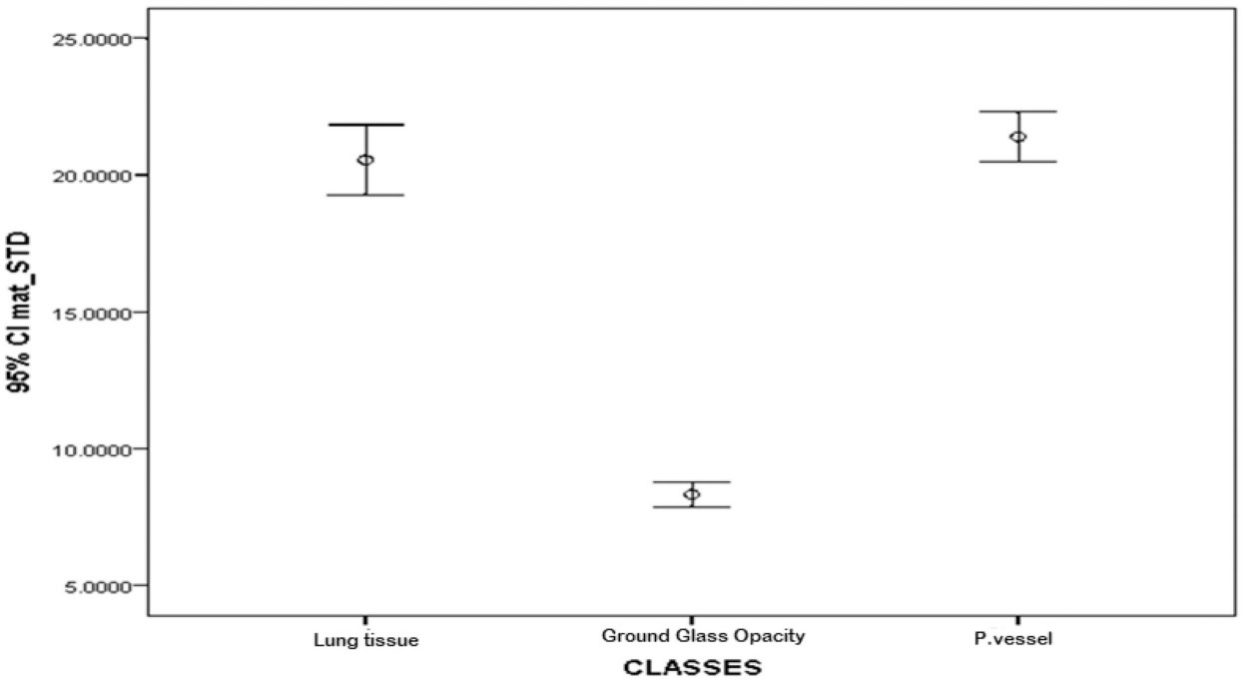
Error-bar plot (mean ± 95% CI) of GLCM standard deviation (SGLD matrix SD) by class—parenchyma, ground-glass opacity (GGO), and intrapulmonary vessels—computed at the ROI level (20 × 20-pixel window). The values are lower for GGO and higher for parenchyma and vessels, matching the class differences seen in Figures 4 and 5.

**Figure 7:**
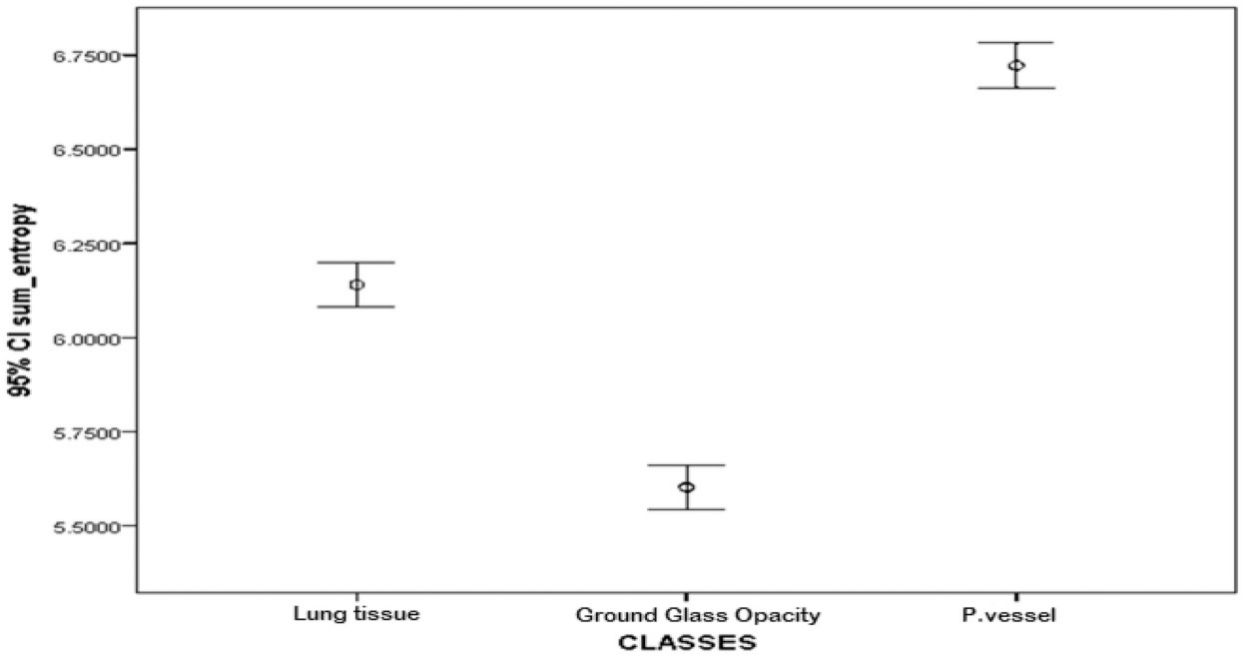
Error-bar plot (mean ± 95% CI) of sum entropy by class, i.e., parenchyma, ground-glass opacity (GGO), and intrapulmonary vessels, computed at the ROI level (20 × 20-pixel window). GGO shows the lowest entropy, parenchyma is intermediate, and vessels show the highest.

**Figure 8:**
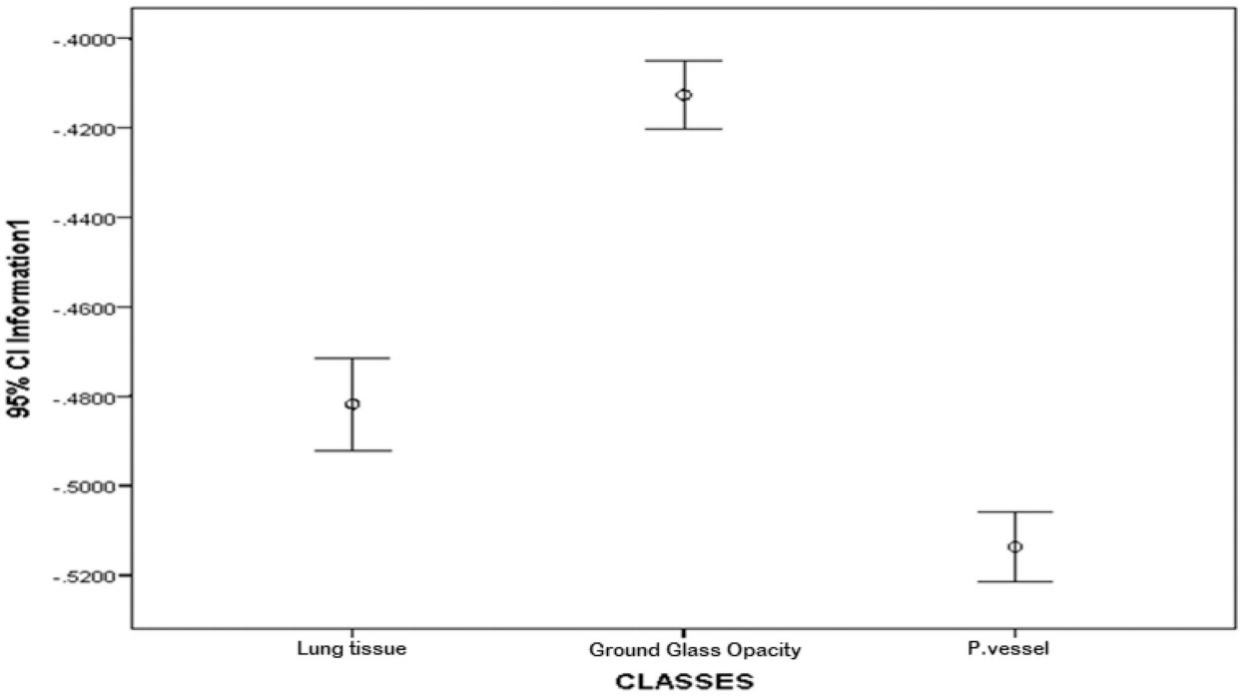
Error-bar plot (mean ± 95% CI) of information correlation measure 1 (IC1) by class, i.e., parenchyma, ground-glass opacity (GGO), and intrapulmonary vessels, computed at the ROI level (20 × 20-pixel window). IC1 is highest for GGO, intermediate for parenchyma, and lowest for intrapulmonary vessels, providing complementary separation to Figures 4–7.

The textural feature average difference clearly distinguished between the texture of ground glass opacity and the intrapulmonary vessels and lung tissue, as depicted in Figures 4 and 5, which illustrate the average difference and inverse difference moment (IDM) for the textural feature within each class, respectively. This distinction was characterized by a minimal standard deviation, with Figures 6–8 illustrating the SGLD matrix standard deviation, sum entropy, and the information correlation measure 1, respectively. Furthermore, the feature sum entropy (Figure 7) effectively differentiated the intrapulmonary vessels and lung tissues from ground glass opacity. Combining these five features reduced the between-class variance while increasing the within-class variance to optimize the classification accuracy and minimize errors.

The classification errors predominantly arose from overlapping textural areas near the borders when the extraction window covered two classes. The discriminatory power of the texture diminished as the window size decreased, potentially leading to the inclusion of other textures at the margins. Thus, the 20 × 20-pixel window consistently demonstrated the highest classification accuracy.

## 4 Discussion

This study presented a clear differentiation of textural features between GGO, intrapulmonary vessels, and lung tissue on high-resolution CT scans. The textural feature average difference effectively distinguished the texture of GGO from intrapulmonary vessels and lung tissue, as indicated in Figure 4 (difference-average by class); Figure 5 (inverse difference moment, IDM); Figure 6 (GLCM standard deviation); Figure 7 (sum entropy); and Figure 8 (information correlation 1, IC1). This differentiation was achieved with the average difference, inverse difference moment, GLCM standard deviation (SGLD matrix SD), sum entropy, and information correlation measure 1. Sum entropy was lowest for GGO, intermediate for parenchyma, and highest for intrapulmonary vessels, although some interference was noted between intrapulmonary vessels and lung tissues for the other four image features.

This approach could serve as a supplementary tool during the radiological interpretation of chest CT scans to reduce classification accuracy errors and enhance diagnostic capability.

Compared with recent AI-based segmentation methods, our approach offers distinct advantages in terms of interpretability, lower data dependency, and potential for routine clinical integration. While deep learning models often require large annotated datasets and operate as black boxes, our method yields transparent, ROI-level outputs that can support radiological workflows with minimal computational burden (13, 14).

The patient-level cross-validation scheme (with feature selection performed within folds) reduces optimistic bias and the risk of overfitting from stepwise procedures, as a patient's ROIs are evaluated on models that were not trained on that specific patient. In addition, the balanced class counts (∼200 ROIs per class) and small final feature set support this study’s statistical robustness regarding the number of observations.

This analysis focused on ROIs and utilized different definitions of texture to describe it as an attribute of an image window (15, 16). Previous studies have emphasized the importance of texture in characterizing objects or regions of interest, with statistical features developed for classifying pictorial images based on pixel intensity.

Using computed tomography texture analysis parameters, including feature extraction with and without filtration, such as SD, MPP, and kurtosis, can assist in distinguishing between patients who are positive or negative for COVID-19 with enhanced specificity compared to basic visual assessment (17).

Texture analysis for computer-aided diagnosis (CAD) has been extensively studied across different medical disciplines, including the diagnosis of breast cancer in mammograms (18, 19), lung nodules in chest radiographs (20–23), osteoporosis in bone x-ray images, and abnormalities in the kidneys and liver. Over the past decade, there has been a significant rise in the utilization of computed tomography texture analysis in oncological imaging, focusing on evaluating the heterogeneity and aggressiveness of solid tumors (24, 25). In thoracic oncology, texture analysis has demonstrated potential in predicting survival (26), assessing the patient’s response to antiangiogenic chemotherapy and immunotherapy in lung cancer (27, 28), distinguishing between lung cancer recurrence and postradiation fibrosis (29), and evaluating the aggressiveness of pulmonary subnodules (30).

Texture analysis has also been applied in diffuse lung diseases, such as pulmonary emphysema, pulmonary idiopathic fibrosis, and pulmonary embolism (31, 32). Specifically, the application of texture analysis on pulmonary angiograms has been effective in providing correlates for ventilated and vascular lungs, aiding in the diagnosis of pulmonary embolism in the presence of other causes of altered vascularity such as emphysema (32). Subsequent studies have investigated the potential of texture analysis to differentiate between diffuse pulmonary alterations that may appear similar on visual assessment. In a study by Kloth et al., texture analysis was shown to be capable of distinguishing active alveolitis from lung fibrosis in patients with systemic sclerosis (33), addressing the diagnostic complexities that arise in visual assessment due to the similar imaging features of the two conditions in their early stages. Therefore, texture analysis could potentially serve a crucial role in the early differentiation of various pulmonary conditions presenting with GGO, leading to individualized treatments and enhancing patient care.

Another study (34) utilized CT image texture analysis to identify pulmonary abnormalities caused by H1N1 influenza on chest CT scans. The study demonstrated that mean intensity, SD, and non-uniformity could differentiate abnormal regions in patients with H1N1 influenza from those with pulmonary fibrosis, normal lung conditions, and non-influenza infections. In line with previous research, our study identified the texture metrics that were key distinguishing factors (correlation information measure 1, IDM, and sum entropy). These metrics captured the heterogeneity (variation in intensity, irregularity, and tissue contrast) within the lung tissue distribution. This study illustrates how CT image texture analysis extracts subtle image characteristics imperceptible to the naked eye, potentially assisting radiologists in their diagnostic tasks.

As highlighted in another study, CT image texture analysis can be utilized to swiftly differentiate COVID-19 from other infectious pneumonias. Moreover, radiomics has been shown to assist in differential diagnosis on HRCT—for example, a radiomics-based model accurately differentiated COVID-19–related ground-glass opacities from those due to other acute lung diseases—and lesion severity grading models have also been reported (mild vs. moderate/severe), supporting the use of complementary quantitative assessments alongside visual reads (35, 36).

Our study illustrates the potential of CT image texture analysis in managing patients with COVID-19. The overall classification accuracy was 88.6%, as depicted in Figure 2, which supports its use as a supplementary tool alongside visual assessment. CT image texture analysis can be readily integrated as a supplementary tool in routine clinical practice, pending confirmation of our initial findings on a larger scale. Our results may reflect potential histopathological disparities in parenchymal findings; the distinct inflammatory infiltrate triggered by COVID-19 could account for the variations observed in the CT image texture analysis among the patients.

The integration of CT imaging texture analysis could enhance the importance of chest CT scans in the setting of COVID-19, potentially optimizing patient care in the emergency department (34).

While the study presented promising findings, it is important to acknowledge certain constraints associated with its retrospective nature, its single-center design, the small sample size, and the lack of an interreader agreement analysis due to the consensus agreement. However, previous studies have demonstrated good reproducibility for texture analysis of filtration-histogram-based CT images, using multicenter clinical validation (26, 37) and demonstrating robustness to variation in image acquisition factors (38, 39). It is worth noting that the GGO ROIs were drawn on a single axial slice. Although a multislice or volume delineation of GGO would be a better representation within the whole lung, such methodology is time-consuming and therefore not practical in the clinical setting. Moreover, studies have indicated similar results in assessing heterogeneity when comparing cross-sectional area analysis to whole-volume analysis on CT scans (40). Finally, the lack of histopathological correlation hindered the validation of the hypothesis regarding the significant parameters in the texture analysis of CT images and the tissue/parenchymal changes induced by SARS-CoV-2 infection and the immune system's response.

### 4.1 Clinical implications

The texture descriptors derived from HRCT patches (e.g., average difference, inverse difference moment, SGLD matrix SD, sum entropy, and information correlation measure 1) separated normal parenchyma, GGO, and intrapulmonary vessels with an overall accuracy of 88.6% when using a 20 × 20-pixel window. In practice, these cues could (i) supplement visual reads when GGO is subtle or confluent; (ii) provide quantitative estimates of GGO burden to support reporting consistency and longitudinal comparison; and (iii) assist in training and auditing, providing interpretable features that align with the radiologists’ mental models rather than a black-box output. Given that the workflow is non-AI and based on interpretable first/second-order statistics, it is feasible to integrate alongside routine HRCT interpretation (e.g., as an ROI-level adjunct) without changing reporting systems. We emphasise that this method is not a diagnostic test for COVID-19; rather, it augments chest CT assessment in patients who already have clinical/virological context.

### 4.2 Study limitations and future work

This analysis used two-dimensional ROIs on a single axial slice, which may underrepresent three-dimensional lesion heterogeneity; however, a prior study showed that largest-slice analyses can approximate whole-volume texture metrics in some contexts (40). Radiomic/texture features are sensitive to acquisition and reconstruction parameters (e.g., slice thickness, kernel, algorithm), which can affect the absolute values and model transferability (4, 41, 42). Our HRCT parameters (including slice thickness) should therefore be viewed as part of the model definition, and future validation should include multiple scanners, multi-center cohorts, and harmonization strategies. Stepwise LDA, while interpretable, can overfit if not carefully cross-validated; external validation and preregistered analysis plans would strengthen the generalizability of the results. The ROIs were created by consensus and interreader agreement was not quantified, which should be addressed in future work with independent readers. Furthermore, moving from ROI-level classification to volumetric, lesion-aware segmentation and assessing the reading time and interreader consistency effects will clarify how best to deploy these features in clinical pathways.

The present dataset only comprised RT-PCR-confirmed COVID-19 cases, focusing on intrapatient tissue-class separation of normal lung parenchyma, GGO, and intrapulmonary vessels. Differentiation between COVID-19 and non-COVID-19 ground-glass opacities was beyond the scope of this study but remains a planned future extension to evaluate this method’s diagnostic specificity and clinical applicability. Although the present work centered on texture differentiation of GGO in confirmed COVID-19 cases, COVID-19 pneumonia may also present with concurrent fibrotic or reticular changes, such as honeycombing, within the same HRCT images. Future research will extend this framework to delineate these coexisting parenchymal patterns, potentially supporting longitudinal assessment of disease progression and fibrotic evolution.

## 5 Conclusion

This study concludes that texture-based feature extraction can aid in classifying lung tissue on HRCT with an overall accuracy of 88.6%, and has potential as a supplementary tool during radiological interpretation of chest CT.

## Data Availability

The raw data supporting the conclusions of this article will be made available by the authors, without undue reservation.
