# Supplementary material for: Texture analysis improves lung-tissue segmentation on high-resolution computed tomography in COVID-19
Source: Front Radiol. 2025 Dec 5;5:1694478. doi: 10.3389/fradi.2025.1694478 (PMC12714659; doi:10.3389/fradi.2025.1694478)
Supplement: Supplementary file 1 [file Table1.docx]

| **Class** | **Sensitivity** | **Specificity** | **PPV** | **NPV** | **F1-score** |
| --- | --- | --- | --- | --- | --- |
| Parenchyma | **85.9%** | **96.5%** | **92.4%** | **93.2%** | **89.0%** |
| GGO | **90.0%** | **92.4%** | **85.8%** | **94.8%** | **87.9%** |
| Intrapulmonary vessels | **89.9%** | **94.0%** | **88.1%** | **94.9%** | **89.0%** |
| **Macro-average** | **88.6%** | **94.3%** | **88.8%** | **94.3%** | **88.6%** |

**Table S1:** Demonstrate ROI-level, cross-validated performance by class (parenchyma, ground-glass opacity, intrapulmonary vessels). Metrics were computed from **out-of-fold predictions** under **patient-level, stratified k-fold cross-validation** and include **sensitivity, specificity, PPV, NPV, and F1-score**. Class totals: parenchyma = 198, GGO = 201, intrapulmonary vessels = 198 (total = 597; correctly classified = 529).
